# Unveiling Hidden Canals: Middle Mesial Canal Prevalence in Mandibular First Molars Using Cone-Beam Computed Tomography Analysis in Central India

**DOI:** 10.7759/cureus.45944

**Published:** 2023-09-25

**Authors:** Shriya R Singi, Anuja Ikhar, Akash Sibal, Amit Reche, Priyanka P Madhu

**Affiliations:** 1 Department of Research and Development, Jawaharlal Nehru Medical College, Datta Meghe Institute of Higher Education and Research (Deemed to be University), Wardha, IND; 2 Department of Conservative Dentistry and Endodontics, Sharad Pawar Dental College and Hospital, Datta Meghe Institute of Higher Education and Research (Deemed to be University), Wardha, IND; 3 Department of Public Health Dentistry, Sharad Pawar Dental College and Hospital, Datta Meghe Institute of Higher Education and Research (Deemed to be University), Wardha, IND

**Keywords:** cone-beam computed tomography, failed root canal treatment, root canal treatment, isthmus, mandibular molar, middle mesial canal

## Abstract

Background

The most frequent reason involving failure of root canal treatment in molar teeth is the inability to locate additional canals. While much research has been conducted on the morphology of mandibular molars, little is known about isthmuses and middle mesial (MM) canals in the Indian population. The primary aim of the present research was to determine the prevalence of MM canals and isthmuses of mandibular first molars in the Central India population using cone-beam computed tomography (CBCT) images.

Methodology

CBCT of 140 mandibular first molar teeth was analyzed from the institutional database images in the coronal, sagittal, and axial planes. Data concerning the number of root canals, the presence of middle mesial canals, and the presence of isthmus were noted. Information collected was transferred into an Excel sheet and analyzed using the Statistical Package for Social Sciences (IBM Corp., Armonk, NY, USA).

Results

Eight images (5.71%) revealed the presence of middle mesial canals with no statistically significant occurrence. The incidence of isthmi in the mandibular first molar was 84.3%, and the presence of isthmi in the coronal third was about 38.6%. This showed a considerable presence.

Conclusion

There is incidence of the middle mesial canal, which emphasizes the need to locate extra canals in patients undergoing root canal endodontic treatment. The implementation of CBCT will help identify the extra canal before the treatment. This will help ultimately in providing better patient care.

## Introduction

Debridement and sealing of the complete root canal space are required for effective root canal treatment [1,2]. If the dentist fails to debride pulp tissue completely, properly disinfect and obturate root canals, or misses noticing the presence of an additional root canal, then in such instances, the entire treatment may fail, leading to clinician and patient dissatisfaction. It is crucial to have knowledge related to various types of root canal morphologies, as it can help locate and negotiate the canals that ensure proper treatment. The most common reasons for failure of endodontic treatment include variations in canal anatomy, insufficient canal instrumentation, and obturation. Furthermore, root canal shape differs significantly between populations and even among individuals in the same community [3-5]. Thus, these variations in canal anatomy are more likely to be responsible for endodontic flare-ups, re-treatment, and failures [6]. Therefore, it is crucial to include imaging as mandatory for preoperative evaluation to understand the root morphology and canal occurrence for better treatment outcomes. 

In dental practice, permanent mandibular molars are among the most frequent teeth that require root canal therapy. They play an important part during chewing, so sustaining them healthy is essential for efficient mastication. The mandibular molars frequently have two roots - mesial root and distal root. Still, among a small proportion of people, a third root emerges [7,8]. During routine diagnosis, the intraoral periapical film frequently overlaps the distal root's image and is usually unclear [9]. If the dental surgeon fails to recognize the canal in the third root, it may go untreated, resulting in endodontic treatment failure.

Mandibular molars' mesial roots typically have a mesiobuccal (MB) canal and a mesiolingual (ML) canal, while their distal roots usually have a single centrally situated canal. The isthmus is a slight connection among the two mesial root canals containing pulp tissue. Treatment success rates were lower for teeth with a prepared isthmus than for teeth without an isthmus, indicating the need to examine isthmus morphology for every tooth in order to better comprehend and monitor surgical procedures and patient outcomes [10]. Variations in anatomy can occur in the isthmus area, which can include the existence of an extra canal known as the middle mesial canal (MMC). Moreover, there may be an additional canal in the distal root [11]. Given that the missing canals might have organic substrates, tissue remnants, or necrotic debris that encourage the spread of microorganisms, it is critical to find all canals, particularly MMC and distolingual root canals, thoroughly debride them, and avert reinfection to avoid endodontic failure during treatment. The anatomic location and morphology of the MM canal vary, and its incidence is affected by age group, ethnicity, and the various study techniques used in the research investigations [12].

Researchers have used several study types, such as root canal treatment procedures, radiographs of root canal treated teeth, analysis of extracted teeth, and in-vitro radiography and sectioning, to document studies on the prevalence of multiple roots, number of canals, and complex canal shape [13]. Dissimilarities in anatomic studies previously identified in the literature may be attributed to variances in age, race, gender, inadvertent bias in case selection, and in-vitro or in-vivo study designs, among other things [14,15].

Since the last decade, the adoption of cone-beam computed tomography (CBCT) scans as a device for diagnosis and treatment planning has received a lot of consideration in the field of dentistry. Computed tomography (CT) scan's ability to analyze pathologic diseases and morphological structures in a three-dimensional form has shown to be extremely valuable. Moreover, CBCT is superior to conventional periapical films in terms of reducing or eliminating superimposition of surrounding structures [16]. It's also difficult to miss the important features of dental CT scans over traditional medical CT scans. This non-invasive scanning has a variety of uses in dentistry, including detailed morphologic analysis, which resulted in a lot of studies on permanent molar teeth that demonstrated that CBCT pre-evaluation helps determine canal configurations. This research aims to identify the prevalence and analyze the configuration of an isthmus and/or true middle mesial canal in the root of mandibular first molars using cone-beam computed tomographic scans in the Vidarbha region of the Central India population.

## Materials and methods

Study setting and design

To determine the prevalence of the middle mesial canal (MMC) in mandibular first molars using CBCT in the Central India population, a cross-sectional study was performed. It was commenced after being approved by the Institutional Ethical Committee of Datta Meghe Institution of Higher Education and Research with reference number IEC/2022/966 [DMIMS (DU)]. From the patient's data bank in Oral and Maxillofacial Radiology within the institute, previous records of the patients who underwent CBCT from January 2020-December 2021 were collected. A general, written, and well-informed consent was obtained from the participants for the use of their data in the studies conducted at the Radiology Centre of the institute.

Sample size calculation

Based on a previous study [17], sample calculation was performed using 95% confidence intervals as N = [Z(1- α/2)]^2 *P*Q/(D)^2, α: Type I Error at 5% level of significance (l.o.s.) = 1.96, β: Type II Error at 20% (1-β) = 0.8, Estimated Proportion (P) =4.5%=4.5, Estimation of Error (D) = 4%, N = 1.96 * 0.045*(0.955)/(0.05)2=104. Approximately 104 cases were needed to have a precision of 4%. The sample size was later increased, and around 140 cone beam data sets were examined of permanent mandibular first molar teeth in 71 males and 69 females.

Study inclusion and exclusion criteria

In the inclusion criteria, permanent mandibular first molars were included in the study. The age of the participants needed to be 18 years or older, regardless of gender, as both males and females were eligible to participate. In the exclusion criteria, cases with unerupted permanent mandibular first molar teeth, root resorption, coronal caries, incomplete root formation, or those teeth that had undergone root canal treatment were excluded from the study.

Methodology

All images were captured with a Planmeca ProMax 3D Classic CBCT unit (Planmeca, Helsinki, Finland) with an image voxel size of 200 um along with exposure parameters of 60-90 kV and 6-15 mA. Images were viewed in a dimly lit environment using Planmeca Romexis Viewer on a Dell AIO Inspiron 5410 Desktop Computer with a 23.8-inch light-emitting diode screen at 1920 x 1080 pixels resolution. The scans have been optimized for the most effective viewing using the built-in features and assessed slice by slice by three examiners in each of the three planes at the same time. If there was a disagreement about the interpretation of a particular CBCT image, the majority of observers' opinions were taken into consideration.

**Figure 1:**
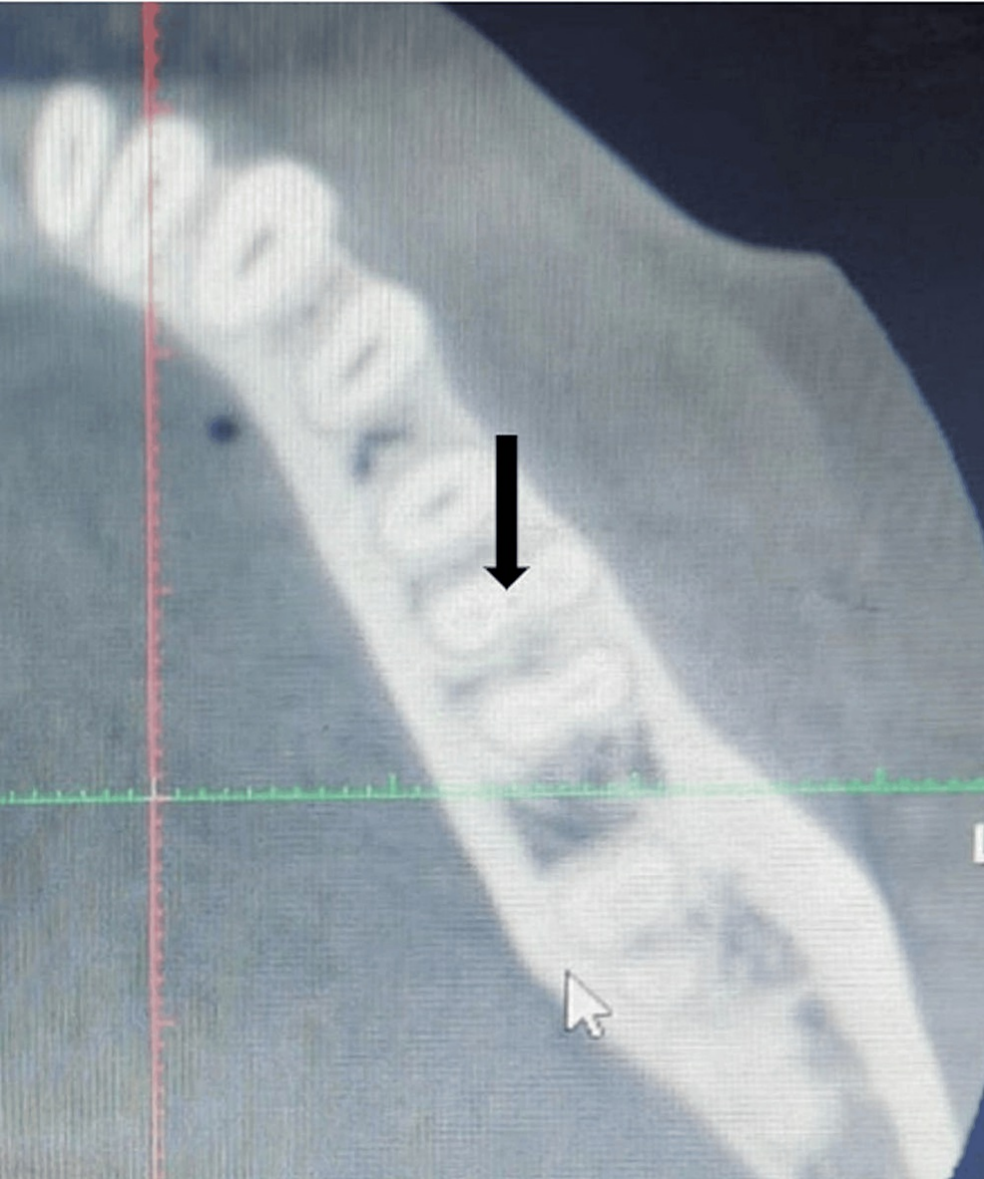
Middle Mesial Canal The black arrow denotes the cone-beam computerized tomographic image showing middle mesial canal with separate canal orifice in axial view.

Furthermore, all teeth were examined from three different perspectives: axial, coronal, and sagittal. During the teeth examination, the number of roots, as well as the number of root canals and their configuration in the mesial root, was recorded. The molar roots also documented the presence and configuration of isthmi and MMC. MMC was identified if a clear round or oval radiolucency was observed between the MB and ML canal in the axial view, regardless of whether an isthmus was present (Figure 1). It was observed that an isthmus existed when there was a narrow ribbon or fin-like connection between the MB canal and the ML canal. If MMC is identified in the axial view, its configuration would be evaluated further in the coronal view using a similar process. The root would first need to be correctly oriented in both the sagittal and axial views. The data was entered into an Excel spreadsheet and analyzed with the Statistical Package for Social Sciences, SPSS software (Version 27.0; IBM Corp., Armonk, NY, USA). The coding for the data found was numbered according to Table 1.

**Table 1:** Coding for Isthmus and Middle Mesial Canal

Code	Isthmus	Code	Middle Mesial Canal
1	Absence of Isthmus	1	Presence of Middle Mesial Canal
2	Presence of Isthmus up to Coronal Third	2	Absence of Middle Mesial Canal
3	Presence of Isthmus up to Middle Third	-	-
4	Presence of Isthmus up to Apical Third	-	-

## Results

A total of 140 CBCT images were analyzed (71 males and 69 females). Of the 140 patients, eight (5.71%) had middle mesial canals (Table 2). In 54 molars (38.60%), isthmi were present in the coronal third, 21 molars (15.00%) were present in the middle third, and 43 molars (30.70%) in the apical third (Table 3). The patients were categorized into four types based on their age and the prevalence of the middle mesial canal was noted in each category. No middle mesial canals were seen in patients <20 years old, six (75%) middle mesial canals were located in patients aged 21-39 years, two (25%) middle mesial canals were found in patients aged 40-59 years, and no canals were found in patients above the age of 60 years. The reported incidence of the MM canal in females was six, whereas in males, it was two, which is 75% vs. 25%, respectively. On examination, around five (62.5%) middle mesial canals were located on the right side of the mandible, and three (37.5%) middle mesial canals on the left side of the mandible. There was no significant association found between the age (P = 0.709), gender (P = 0.134), and side of the mandible examined (P = 0.196) against the prevalence of the middle mesial canal as revealed by the Chi-square test. There was no statistically significant presence of MM canals. However, a statistically considerable presence of isthmi in mandibular molars was seen. It was discovered that 11 mandibular first molars (7.9%) had a third root, known as "radix entomolaris."

**Table 2:** Prevalence of Middle Mesial Canals in Mandibular First Molars

Middle Mesial Canal	Frequency	Percentage
Present	8	5.71%
Absent	132	94.28%
Total	140	100

**Table 3:** Prevalence of Isthmus in Mandibular First Molars

Isthmus	Frequency	Percentage
Not present	22	15.70%
Coronal third	54	38.60%
Middle third	21	15.00%
Apical third	43	30.70%
Total	140	100

## Discussion

The complete disinfection of the pulp canal space is crucial for root canal treatment to be successful. Several studies have been conducted to analyze the anatomy of mandibular molars. Many variations exist, and one such occurrence is the MMC in the mandibular molar. The MM canal is a supplementary canal occurring in the mesial root of mandibular molars, usually seen between the mesio-buccal and mesio-lingual canals. In the groove within both of the main canals, the middle canal orifice can be found beneath a dentinal projection. When a single root extension is unable to close itself off and forms a constriction, an isthmus is formed [17]. The dentin layer in this groove is paler in color than the dentin surrounding it. According to studies, the mandibular first and second molars' grooves have an average length ranging from 1.07-2.81 mm and an average depth of about 1.05 mm and 0.17-7.66 mm [18]. According to Pomeranz et al. [19], the middle mesial canals have been divided into three different types namely independent, fin, and confluent.

Periapical radiography plays a crucial part in endodontic procedures [20], but due to its limitations of being 2D and showing superimposed canals, it can be challenging to locate the middle mesial canal through this method [21]. Using CBCT, Wang et al. (2010) [22] found that 2.3% of the population under investigation had the MM canal. These investigations, however, did not focus solely on MM canals; they also looked at root canal morphology. Therefore, Vertucci's (1984) [23] classification was modified further to analyze the incidence of MM canals. CBCT is currently being employed in endodontics for improved comprehension of the root canal system, and its findings are consistent with the methods used in laboratories [24,25]. CBCT has also been employed in various research to detect the middle mesial canal and the morphological features related to the roots of the mandibular first molar [21]. 3D imaging is done using CBCT, which helps in detecting new canals by analyzing various sections of the canal [21]. Furthermore, most findings have confirmed the occurrence of two canals in the mesial root [2,26,27]. Vertucci and Williams reported the first occurrence of three discrete mesial canals with different foramen and orifices [23].

The middle mesial canal has been observed in people from Asia, Africa, Europe, and even North and South America. Additionally, geographical variations have been noted in the findings of the studies. Significant variations in the incidence of MMC were discovered by Nosrat et al. (2015) [28] between non-White (29.4%) and White (12.2%) patients as well as Turkish and Brazilian populations, respectively. Baugh et al. observed an incidence range of 1%-15% for the middle mesial canal in a retrospective evaluation of this canal in 2004, which is in accordance with the current study's findings [20].

The research by Tahmasbi et al. investigated the morphological configuration of the mandibular molar with the help of cone-beam computed tomographic to find the occurrence of a middle mesial canal and/or isthmus in the roots of mandibular molars. A sample size of 122 mandibular molars was evaluated and according to the results, twenty (16.4%) of the 122 teeth had the presence of middle-mesial canals. The results showed that 26% of first molars and 8% of second molars (P<.05) had MM canals in them [29]. Another study was executed by Sajjan et al. in the Andra Pradesh subpopulation of South India and CBCT of 89 patients was chosen at random from the institution's database. The findings of the study showed four images (4.5%) that revealed Middle Mesial canals with no statistically significant occurrence. The occurrence of isthmi in the mesial roots was around 52.7%. This showed a considerable presence. Therefore, to attain better outcomes in endodontic treatment, dental operating microscopes and modern technology should be applied during the cleaning and obturating of isthmuses [17].

These researches are in concurrence with the study done by Hasheminia et al., where a total of 768 radiographs of mandibular first molars, 384 each for males and females, were evaluated for the prevalence of MM canal based on the gender of the patients. A Chi-square test was done to correlate the data, and it was considered statistically significant (P < 0.05). A mid-mesial canal was found in 24 samples (3.13%). The reported incidence of the MM canal in males was 15, whereas in females, it was nine, which is 3.92% vs. 2.35%, respectively. No statistically significant difference was found (P = 0.21), concluding the occurrence of MM canals associated with gender. As a result, the study suggests that additional canals in individuals undergoing endodontic treatment should be detected.

In endodontic procedures, management of root canal isthmus is critical [30]. Complete biomechanical preparation and obturation of the apical third of root canals are believed to be among the most significant variables for attaining a favorable outcome with root canal treatment. An unprepared isthmus, particularly in the maxillary and mandibular molars, may contain tissue remnants and necrotic debris that act as a source for the accumulation of bacteria, resulting in endodontic treatment failure [30,31]. The challenges encountered in the biomechanical preparation of the mesiobuccal root canal system during root canal procedures may fail and might necessitate the need for re-treatment. As a result, basic anatomical knowledge, isthmus recognition, and management may be highly beneficial in increasing the success rate of endodontic procedures in posterior teeth [32-34].

There is a link between apical periodontitis and untreated canals and isthmi, according to research [35,36]. In untreated canals and isthmi, biofilm can form, and the canals and isthmi can get clogged with microbes. In addition, disinfecting irrigants would only reach these locations if they were instrumented [36]. Even though the irrigant enters these areas, it may not be enough to remove the biofilm [36]. Therefore, the instrument must thoroughly irrigate these regions regardless of an MM canal/isthmus presence.

Although CBCT is an excellent diagnostic tool for detecting middle mesial canals, clinicians must note that it exposes the patient to additional radiation. Therefore, it should be used only in those cases where its need can be justified clinically. The financial cost of using CBCT must also be noted as it would be an added cost for treatment. Moreover, due to lack of availability of CBCT machines at dental centres and inability to accurately detect additional canals in periapical radiograph, it is not possible to locate the MMC in patients undergoing root canal treatment. As a result, an attempt should be made to make CBCT available in dental centers. In addition, CBCT is not used in all cases of root canal therapy and only taken into account when additional radiographic details are required for diagnosis and treatment planning. The study's limitations include the possibility that the population studied was not representative because it was conducted in a single location.

## Conclusions

The prevalence of middle mesial canals is rare, about 5.71% in the subpopulation of Central India. The most frequent cause of failure in endodontic treatment of mandibular molars involves the presence of a canal isthmus. Careful examination of the area between canal orifices is required to avoid missing the MMC, which would result in unfavourable clinical outcomes. Because the MM canals or isthmus at the apex of the root can operate as portals of exit, identifying and debriding these sites during endodontic treatment is essential. The use of CBCT will help in the identification of the extra canal before the treatment. This will help in providing better patient care.
